# Automated fluid delivery from multiwell plates to microfluidic devices for high-throughput experiments and microscopy

**DOI:** 10.1038/s41598-018-24504-x

**Published:** 2018-04-18

**Authors:** Ross C. Lagoy, Dirk R. Albrecht

**Affiliations:** 1 Department of Biomedical Engineering, Worcester Polytechnic Institute, 100 Institute Road, Worcester, MA 01609 USA; 2 Department of Biology and Biotechnology, Worcester Polytechnic Institute, 100 Institute Road, Worcester, MA 01609 USA

## Abstract

High-throughput biological and chemical experiments typically use either multiwell plates or microfluidic devices to analyze numerous independent samples in a compact format. Multiwell plates are convenient for screening chemical libraries in static fluid environments, whereas microfluidic devices offer immense flexibility in flow control and dynamics. Interfacing these platforms in a simple and automated way would introduce new high-throughput experimental capabilities, such as compound screens with precise exposure timing. Whereas current approaches to integrate microfluidic devices with multiwell plates remain expensive or technically complicated, we present here a simple open-source robotic system that delivers liquids sequentially through a single connected inlet. We first characterized reliability and performance by automatically delivering 96 dye solutions to a microfluidic device. Next, we measured odor dose-response curves of *in vivo* neural activity from two sensory neuron types in dozens of living *C*. *elegans* in a single experiment. We then identified chemicals that suppressed optogenetically-evoked neural activity, demonstrating a functional screening platform for neural modulation in whole organisms. Lastly, we automated an 85-minute, ten-step cell staining protocol. Together, these examples show that our system can automate various protocols and accelerate experiments by economically bridging two common elements of high-throughput systems: multiwell plates and microfluidics.

## Introduction

Microfluidic devices offer several advantages for biomedical research, particularly for presenting precise physical and chemical environments to cells and organisms^1–4^, multiplexing experimental conditions^4–6^, and reducing reagent volumes for screening applications^7–9^. Traditional high-throughput screening systems use multiwell plates to contain samples and chemicals in miniaturized wells and observe each condition after static long-term exposure periods, typically hours to days. For dynamic chemical delivery and monitoring, such as stimulation with brief pulses of bioactive compounds, few options exist to directly interface chemicals in multiwell plates with microfluidic devices. Current approaches include complex setups with separate inlet tubes for each well (i.e., 96 inlet tubes for a 96-well plate)^5^, the use of conventional liquid-handling robots to inject liquids to device inlets^10,11^, and microfluidic designs that are integrated into plastic multiwell dishes^12–16^. These approaches are generally expensive, either due to laborious fabrication processes or to single-use cartridges operated by specialized flow control equipment. A simple yet laborious alternative is the manual transfer of an inlet tube from well to well^17^. However, any disconnection of tubing tends to introduce air bubbles due to surface tension, and these bubbles can severely disrupt fluid flow within microfluidic circuits. Rotational valves can change inlet streams without introducing bubbles, but are generally limited to 8 or 12 positions, while in-line debubblers can remove air from tubing, but occupy relatively large fluidic volumes, and both can be expensive and difficult to clean.

In this paper, we present a robotic system that reliably and automatically transfers a single microfluidic inlet tube from one fluid reservoir to another, without introducing a bubble. In this manner, numerous liquids can be delivered sequentially into any microfluidic device. We demonstrate that this system, based on inexpensive open-source hardware and software, can completely automate: (1) the measurement of neural dose responses and optimization of chemical concentrations for robust and non-saturating responses, (2) a complex chemical screen to determine the effect of different solvents on optogenetically-activated neural responses in living nematodes, and (3) a multi-step, multi-duration cell staining protocol. In each example, the experiment was first set up by preparing a multiwell plate with desired chemicals, preparing the microfluidic device with bioassay targets (e.g. organisms or cells), then selecting the chemical exposure time course and data acquisition settings. Afterwards, all subsequent experimental activity required no user intervention. We expect that this versatile tool will expand the throughput of biological experiments that require serial delivery of multiple fluids, from neuronal imaging in living organisms to histochemical staining of cells. Further, such automation can improve results and reproducibility by minimizing human error and by simplifying the optimization of experimental protocols.

## Results

### A robotic platform to interface multiwell plates with microfluidic devices

**Figure 1:**
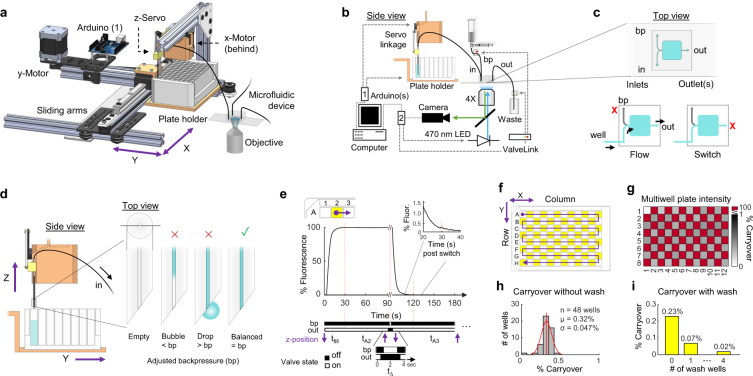
Robotic fluid delivery components, operation principle, and characterization of switching performance and well-to-well carryover across a 96-well plate. (**a**) Model of the robotic platform comprised of a multiwell plate holder with x-y motion and a servo linkage with vertical z-motion to raise and lower an inlet tube connected to a microfluidic device mounted on a microscope stage (Supplementary Video S1). (**b**) Control schematic of the robotic platform, microfluidic device valves, and microscope optical components. Device connections include an inlet (in) tube, backpressure (bp) reservoir, valve, and tube, and an outlet (out) valve and tube. Robotic positions are computer-controlled by Arduino (1) and all valves and microscope illumination are controlled by Arduino (2). (**c**) Top view of an example microfluidic device with a multiwell plate inlet (in), and backpressure (bp) port, and one waste outlet (out). Valve states (red X, closed) are illustrated below during well flow (left) and well-to-well switching (right). (**d**) Side view of the raised inlet tubing and magnified views demonstrating proper backpressure (bp) balance: bp too low can introduce an air bubble in the inlet tubing whereas bp too high can cause backward flow and a drop to emerge from the inlet tubing. (**e**) Timing of valve actuation and servo motion during well-to-well transfer and dynamics of the fluid switch monitored with fluorescein dye. Upper graphs show the time course of relative fluorescence intensity beginning with the switch from a buffer well to fluorescein at t = 0, and a switch back to buffer at t ≃ 90 s. Inset shows a magnified view from 110–130 s (i.e. 20–40 s after the second tube switch). Below, corresponding actuation states of backpressure (bp) and outlet (out) valves and inlet tube z-position (purple arrows) during the short (~2 s) transition time (t_Δ_). Fluid switching in the microfluidic device is complete after a filling delay (t_fill_, vertical red lines) dependent on flowrate and inlet tube volume. (**f**) Multiwell plate map showing 96 alternating buffer and fluorescein dye wells scanned in a “snake” pattern. (**g**) Heat map shows percent carryover without wash steps from fluorescent intensity in the microfluidic channel, calculated for each buffer well relative to the prior fluorescein well after a fill delay of 30 s. (**h**) Histogram of well-to-well carryover without wash steps across 48 wells, from data shown in g. (**i**) Sequential tubing wash steps further reduce well-to-well carryover. Data are shown using a fill delay of 30 s.

The robotic system automates serial liquid delivery from standard multiwell plates to microfluidic devices by automatically raising and lowering an inlet tube into the desired well of a multiwell plate (Fig. 1a). It uses commercially-available parts, machined or 3D-printed brackets, and custom open-source software (Fig. 1a, Supplementary Figs S1, S2), keeping costs below $500. Also, the modular system mounts to a microscope for visualization and monitoring of samples contained within an integrated microfluidic device. The robot uses stepper motors to position the multiwell plate and a servo motor to raise and lower the inlet tube with sufficient force to reliably puncture plate sealing film (Fig. 1a–c, Supplementary Video S1). The system works with both reversibly-sealed and permanently-bonded microfluidic devices and can be adjusted for different plate configurations, such as 6-well to 384-well plates with shallow or deep wells.

To prevent a bubble from entering the robotic inlet tubing when it is raised, flow through the device is momentarily stopped by closing the outflow valve. Capillary pressure at the open end of the inlet tube can be exactly counteracted by a small hydrostatic pressure supplied from a backpressure reservoir elevated above the multiwell plate. In this pressure-balanced configuration, the inlet tube can safely lift out of a well without forward fluid flow (which would introduce a bubble into the inlet tube) or backward flow (which may transfer some inlet liquid to the next well) (Fig. 1b–d).

The mechanical transfer of inlet tubing from one well to another is rapid, requiring less than 2 s transition time to raise the inlet tubing, move the plate, and lower the inlet tubing into the next well (Fig. 1e). However, each new fluid flows sequentially through a single inlet, and therefore must flush the prior fluid from the inlet tube before reaching the microfluidic device. To minimize this switching delay, tubing was as short and narrow-bore as practical (22 cm, 250 µm inner diameter, containing about 11 µL volume). At a flowrate of 2 µL/s, about 30 s were required to completely replace the prior fluid, due to Taylor dispersion within the inlet tube (Fig. 1e, Supplementary Fig. S3); hence, about 5 inlet tube volumes must flow before the fluid switch is complete. To measure reliability and well-to-well fluid carryover, we monitored concentration profiles during fluid switches across a full 96-well test plate containing alternating fluorescein dye and water solutions (Fig. 1f–h). Using a 30 s fill delay and 2 µL/s flowrate driven by hydrostatic pressure, well-to-well carryover was 0.32% ± 0.047% S.D. across all 48 dye-to-water fluid switches. Additional wash steps reduced carryover to less than 0.02% (Fig. 1i).

### Automatic determination of chemical dose responses in *C. elegans* sensory neurons

**Figure 2:**
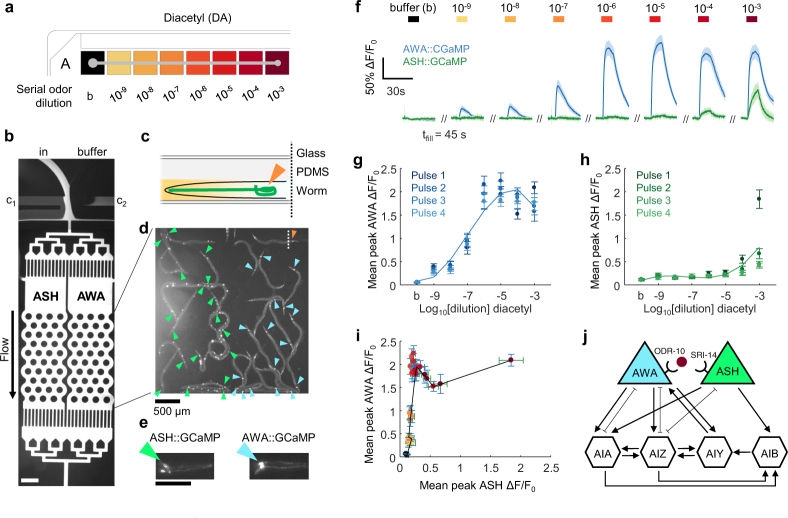
Automated chemical dose response of two chemosensory neurons in *C*. *elegans* to the odor diacetyl from a single experiment. (**a**) Schematic of the multiwell plate prepared with buffer (b) and serial dilutions of diacetyl odor, from 10^−9^ (11.5 nM) to 10^−3^ (11.5 mM). Gray dot indicates the first well position. (**b**) A reusable two-arena pulse microfluidic device with four fluidic inlets, two worm-loading ports, and an outlet. The device switches between the stimulus inlet (in), shown with high concentration fluorescein, and unlabeled buffer, depending on which of two control channels (c_1_, c_2_) are flowing (Supplementary Video S2). Here, c_1_ flows while c_2_ is closed, such that stimulus fluid flows through the animal arenas. Scale bar, 500 µm. (**c**) Cross-section schematic of a worm head expressing GCaMP in a single neuron in the microfluidic assembly. Orange arrow points to the fluorescent cell body (soma) as in d. (**d**) Wide-field (4X) fluorescent image of 36 animals expressing GCaMP in either ASH (green arrows, left) or AWA (blue arrows, right) chemosensory neurons in the device. (**e**) Magnified images from panel d of animals expressing GCaMP in either ASH (left) and AWA (right) chemosensory neurons. Arrow points to soma. Scale bar, 100 µm. (**f**) Time course and average normalized fluorescence (∆F/F_0_) dose response traces for AWA (blue) and ASH (green) neuron cell bodies. Four pulses (10 s each) were delivered once per minute at each increasing concentration (or buffer). Lines and shading represent mean and SEM across four pulses, n = 72 traces per concentration and neuron type. (**g**) Mean peak normalized calcium responses for AWA neurons across seven increasing odor steps and buffer (b). Data points show population average and SEM (n = 18) for each of four repeated pulses per concentration. Trace represents a third-degree polynomial fit of each concentration’s pulse average. (**h**) Mean peak normalized calcium responses for ASH neurons, as described in g. (**i**) Mean peak normalized calcium responses of AWA plotted against ASH for each individual odor pulse. Error bars represent SEM for AWA (blue) and ASH (green), per concentration (corresponding dot color). (**j**) Neural network wiring diagram illustrating chemical synapses (arrows) and gap junctions (flat ends) for AWA and ASH sensory neurons and four first layer interneurons. Diacetyl is detected by different affinity receptors on each sensory neuron^19,27^.

Neural dose-response curves identify the sensitivity of neurons to a particular chemical stimulus and determine optimal working concentrations that elicit robust yet unsaturating responses. They have been manually generated in *C*. *elegans* by optical imaging of animals expressing a genetically-encoded calcium sensor during exposure to pulses of multiple concentrations of chemical stimuli in microfluidic devices^17,18^. To simplify this tedious manual process, we set up a completely automated dose-response experiment by filling serially-diluted stimuli in a 96-well plate (Fig. 2a). About 20 young adult *C*. *elegans* expressing GCaMP in either the AWA or ASH chemosensory neurons were introduced into a dual-arena microfluidic device (Fig. 2b–e) and subjected to brief pulses of the odor diacetyl (DA) across six orders of magnitude (11.5 nM to 11.5 mM, 10^−9^ to 10^−3^ dilutions) (Supplementary Video S2). AWA chemosensory neurons showed an increase in calcium upon diacetyl exposure with an EC_50_ around 1.15 µM (10^−7^ dilution) and saturation above 11.5 µM (10^−6^ dilution) (Fig. 2f,g and Supplementary Fig. S4). Activity in the ASH multimodal neurons was not observed until high odor concentrations above 1.15 mM diacetyl (10^−4^ dilution) (Fig. 2f,h). AWA neuron responses were consistent over four repeated odor pulses, whereas ASH neurons adapted rapidly (Fig. 2g,h, Supplementary Fig. S4). These measurements replicated previously-reported results^17,19,20^, and our automated experiment further revealed a negative correlation between ASH activity and AWA peak response magnitude at high odor concentrations (Fig. 2i) that may arise from circuit feedback (Fig. 2j).

### An automated compound screen yields suppressors of stimulated neural activity in *C. elegans*

**Figure 3:**
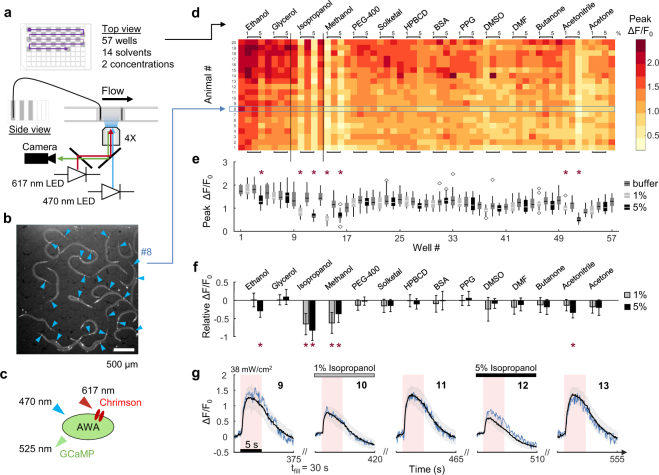
An automated *C*. *elegans* solvent screen yields suppressors of optogenetically-activated neural responses. (**a**) Multiwell plate map showing 57 wells containing solvents at 1% and 5% concentrations alternating with control S. Basal buffer (gray fill). Diagram below shows the optical path for simultaneous stimulation and monitoring of neural responses, *via* 470 nm blue excitation of GCaMP and green emission, and 617 nm red light stimulation of the Chrimson ion channel. (**b**) Wide-field 4X fluorescence image of young adult animals co-expressing GCaMP and Chrimson in the AWA sensory neurons in each animal (arrows) within a reversibly-sealed single-arena microfluidic device. (**c**) A schematic of excitation and emission wavelengths used for simultaneous optogenetic stimulation of AWA sensory neurons using Chrimson and recording of intracellular calcium levels with GCaMP. (**d**) A heat map representing 1,140 peak normalized neural responses (∆F/F_0_) across 20 individual animals and 57 solutions. Horizontal blue box highlights animal #8, identified in panel b. (**e**) Box plot shows population average peak ∆F/F_0_ neural activation response for each compound. Symbols represent statistically significant mean peak differences compared to immediate prior controls (*p < 0.001, ANOVA repeated measures with Bonferroni correction). Boxes indicate 25^th^ and 75^th^ percentiles, whiskers extend to 1.5 times the interquartile range, and outliers are indicated with diamond symbols. (**f**) Bar graphs show the difference in mean peak ∆F/F_0_ response between each solvent concentration and its immediate prior buffer control. Error bars represent SD. Symbols represent statistically significant relative mean peak differences (*p < 0.001, paired one-sample t-tests with Bonferroni correction). (**g**) Example neural traces show suppression of optogenetic stimulation by isopropanol. Population average animal ∆F/F_0_ calcium responses (black line) to 5 s of red light stimulation are shown for wells 9 to 13 (outlined in d). Shading represents SD. Blue lines indicate responses from animal #8.

The robotic system was used to screen fourteen common solvents and carriers for their effects on optogenetically-evoked neural responses (Fig. 3). Each solution was prepared at two concentrations (1% and 5%) in a 96-well plate alternating with buffer control wells for 57 total solutions delivered sequentially to a microfluidic neural imaging device (Fig. 3a). Calcium signals were recorded from AWA chemosensory neurons of 20 young adult animals co-expressing the genetically encoded calcium sensor GCaMP2.2b and the red-shifted channelrhodopsin Chrimson in the same neuron, allowing simultaneous optical stimulation with red light and monitoring of neural calcium responses (Fig. 3b,c). Most light-stimulated neural responses were unaffected by <1 min exposures to each solvent concentration, compared with the buffer control response just prior (Fig. 3d–f, Supplementary Fig. S5). However, isopropanol, methanol, and acetonitrile significantly suppressed peak fluorescent responses at both 1% and 5% concentrations, while ethanol decreased responses only at 5% concentration. Solvent effects were transient, as returning to buffer within <1 min rapidly restored normal responses (Fig. 3d,e,g) in most cases, except after 5% methanol and 5% acetonitrile. Response patterns could be monitored from individual animals, allowing paired comparisons with greater statistical power, and were consistent across the population tested (Fig. 3b,d,g, Supplementary Fig. S5).

### Automated multi-step cell fixation and staining in a microfluidic device

**Figure 4:**
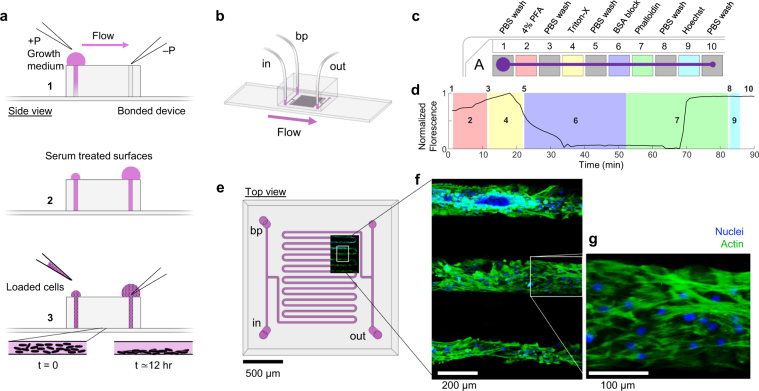
Automated multi-step fixation and staining of cells in microfluidic channels. (**a**) Side view schematic of the microfluidic cell culture preparation by treating surfaces with growth medium (1–2), then loading cells and allowing them to adhere to the glass surface overnight (3, below). (**b**) Schematic of the fluid inlet (in), backpressure (bp), and waste outlet (out) connections for a bonded serpentine channel microfluidic device. (**c**) Ten staining solutions filled in one row of a multiwell plate (A1-A10). PBS, phosphate-buffered saline. PFA, paraformaldehyde. BSA, bovine serum albumin. (**d**) Average fluorescence intensity monitored throughout the entire staining procedure at 1 frame per minute (Supplementary Video S3). Times during each fluid incubation step (1–10) are highlighted. (**e**) A top view of the serpentine channel microfluidic device design overlaid with a low magnification field of view of final stained and monitored area of cells. Inlets and outlets are labeled as in panel b. (**f**) A magnified area of cells from panel e in three microfluidic channels. Green, phalloidin staining of actin; and blue, Hoechst staining of nuclei. (**g**) A magnified area of cells from panel f showing aligned actin filaments and nuclei in a segment of one microfluidic channel.

Histochemical staining is often a laborious process, requiring several manual steps of fixation, washes, blocking, and labeling with visible or fluorescent probes over several hours. We used the robotic system to automate a typical cell staining procedure. Human mesenchymal stem cells (hMSCs) seeded in a microfluidic device were stained one day later by connecting the device to a multiwell plate containing ten wells filled with a sequence of staining solutions (Fig. 4a–c). An 85-min cell staining protocol was entered using a custom MicroManager script which controlled well position, exposure duration, and acquisition of images to monitor staining progression (Supplementary Video S3). Cells were automatically fixed in paraformaldehyde, permeabilized in Triton-X, blocked with bovine serum albumin (BSA), and stained with fluorescent phalloidin to label filamentous actin and with Hoechst to label nuclei (Fig. 4c). For long exposure steps (>30 min), flow through the microfluidic device was paused to prevent inlet wells from running empty and to conserve expensive reagents. Average image fluorescence increased quickly after about 15 min exposure to phalloidin (Fig. 4d), and high-magnification images showed actin filaments aligned within the microfluidic channel (Fig. 4e–g).

## Discussion

The robotic system described in this paper automates sequential delivery of different fluids from multiwell plates to microfluidic devices. Multiwell plates are easily filled manually or by liquid-handling robots, such as for large-scale screens using commercially-prepared compound libraries. After microfluidic device set-up and multiwell plate preparation, complete automation of liquid transfer spares laborious manual effort and eliminates user error, especially for large chemical screens and protocols that require multiple steps. The modular system mounts on a microscope stage for optical monitoring of the microfluidic device throughout the experiment. A custom graphical user interface provides flexibility in protocol design and timing of each chemical presentation, while automatically-generated protocol files can be saved for exact replication of complex experiments. Additionally, the robotic system is compatible with other multiwell plate formats for increased throughput (384 wells, 225 µL capacity), or longer flow durations (e.g., 48 well, 5 mL capacity; 24 wells, 10 mL capacity; 6 wells, 16.8 mL capacity).

Our robotic system compares favorably with other liquid-delivery strategies, outlined in Supplementary Table 1. Rotational distribution valves can interface with large fluid reservoirs and are small enough to mount on a microscope stage, but are limited to 8 or 12 different inlet positions. Autosampling robots can draw liquids from large multiwell plates, but their fluid delivery is limited to the typically small volume contained within the injection needle or syringe. Compared with these commercial devices, the robotic system presented here delivered larger volumes at faster flowrates, achieved a similarly low carryover percentage with wash steps (<0.02%), and could be mounted on a microscope, at substantially lower cost.

For this system, the backpressure design is key to preventing the introduction of air bubbles, which can disrupt microfluidic flow, as the inlet tube is moved from one well to another. Balanced backpressure is readily achieved by raising or lowering a separate inlet reservoir until no air is drawn into the raised inlet tube and no droplet emerges. To implement this strategy, microfluidic devices need at least two inlets, one for drawing liquids from the multiwell plate and another to provide backpressure. Devices that already include multiple inlets need no additional backpressure inlets, such as the neural dose response experiment (Fig. 2), which contained a buffer reservoir that provided sufficient backpressure. While each example here used gravity-driven flow by hydrostatic pressure, the backpressure system is also compatible with vacuum or syringe-pump flow control applied from the outlet tubing.

Reliable fluid switching occurred across all wells of a 96-well plate with an average well-to-well liquid dye carryover of about 0.3% using a fill delay of 30 s, corresponding to about 5 inlet tube volumes. While sufficiently low for most experiments, carryover may be lowered further by increasing the fill delay time, increasing flow rate, or both. Adding wash steps further reduced carryover by an order of magnitude, to the level of commercial autosamplers. However, these changes may reduce overall experimental throughput by slowing fluid transitions, by lowering the available flow time per well, or reducing the number of available fluid wells per plate. For standard lab experiments, we found that the Teflon inlet tubing and glass-coated multiwell plates could be cleaned and reused repeatedly. However, particularly hydrophobic chemicals or sensitive assays may warrant the replacement of all tubing and programming of wash steps, the use of immiscible carrier fluids, and/or extended fill delay times.

We demonstrated three types of automated experiments, each using different microfluidic device designs and different stimulation approaches, and each resulting in increased throughput and decreased manual effort. First, we measured step responses to concentrations of the odor diacetyl across six orders of magnitude in two sensory neuron types from ~40 living *C*. *elegans* in a single experiment, resulting in 1,152 neural calcium traces. In one experiment, our assay replicated previous results from several manual neural imaging assays^17,19,20^, showing that at high odor concentrations above ~1 mM diacetyl, attractive AWA sensory neurons and aversive ASH neurons are simultaneously active. Since diacetyl elicits attractive behavior at moderate concentrations and aversive behavior at high concentrations, ASH signals appear to dominate the behavioral response. However, because ASH neural responses adapt rapidly while AWA responses are consistent, chemotaxis behavior is expected to be transiently aversive. Our results also revealed a negative correlation between ASH activity and AWA peak response magnitude not reported previously, suggestive of negative circuit feedback from ASH to AWA *via* indirect synaptic connectivity between these neurons. This example demonstrates the ability to pulse multiple chemicals in sequence for precisely-controlled durations, as might be required for large-scale chemical stimulation experiments.

Next, we screened a panel of commonly-used solvents and carriers for their effects on stimulated responses in sensory neurons. Most chemical libraries are stored in solvents such as DMSO for solubility and chemical stability, while some show acute suppressive effects on neurons^21^ and chronic exposure can lead to abnormal organism development^22^. In our assay, even brief <1 min exposures of 1% and 5% methanol, isopropanol, and acetonitrile suppressed optogenetically-activated neural activity, as did 5% ethanol. Although acute solvent effects were transient, these results suggest that careful selection of solvents is prudent when screening drug libraries for neural effects. Since activation and neural response were in the same neurons, this observed suppression reflects intracellular interference by these solvents. Equally interesting would be the identification of solvent effects on synaptic communication, by monitoring post-synaptic neuron responses to pre-synaptic stimulation, and evaluating any differences in solvent sensitivity across neural (or other) cell types.

Lastly, we automated an 85 min ten-step cell staining protocol, in which each sequential chemical exposure step lasted a different duration, from 30 s to 30 mins. With a 2 mL deep 96-well plate and a typical flow rate of 2 μL/s, continuous flow lasts about 17 min per well. To achieve longer incubations, flow was paused by closing the outflow and backpressure valves, allowing for unlimited incubation duration and reduced reagent use. Alternatively, if continuous flow was necessary, the inlet could be moved to additional wells containing the same fluid. Notably, the robotic system was effective with a variety of solutions with different physical properties such as surface tension and viscosity (e.g. Triton X-100 and BSA). Further, several important applications would be enabled by automated cell culture and sample processing as shown here. For example, immunohistochemistry with new antibodies typically requires optimization of multiple staining solution concentrations and times, including primary and secondary antibodies and blocking solutions. By automating the staining procedure and live monitoring of imaging results, protocols could be quickly optimized for maximum label specificity and contrast. Additional cell culture applications include the automatic passaging of cells, by timed exposure to buffer, trypsin, and culture medium, or the sorting and collection of specific cell types using additional outflow valves and sample collection tubes.

Together, these practical examples demonstrate the functionality and versatility of our inexpensive, open-source, and customizable platform for interfacing multiwell plates with microfluidic devices. While complex systems exist to introduce multiwell plate fluids though numerous parallel tubes to microfluidic sample chambers^5^, automated serial delivery has the additional advantage of enabling the tracking of individual animal (or cell) responses across many compounds, yielding greater reliability and experimental sensitivity^17^. By automating experimental protocols with systems as presented here, the scientific bottleneck shifts to data analysis, and similar advances in automated data analysis and visualization techniques will be useful for rapid data-driven exploration, protocol optimization, and scientific discovery.

## Methods

### Robotic system components

Two stepper motors (NEMA-17) set the x- and y-position of the multiwell plate holder, using two position limit switches to define the origin, and a servo sets the vertical z-position of inlet tubing. All actuators were controlled by an Arduino Uno R3 with two stacked motor shields (DC Motor Stepper and Servo Shield, Adafruit). The plate holder attached to a linear bearing slide rail for y-motion, which in turn traveled along two parallel V-slot linear rails (OpenBuilds) for x-motion (Fig. 1a, Supplementary Fig. S1). The custom multiwell plate holder was constructed from 3D-printed ABS plastic, or from aluminum to prevent sagging of a full, deep-well 96-well plate, >200 g. The inlet tube moved vertically by a servo assembly comprised of an aluminum or ABS servo mount, a servo arm, and an aluminum linkage connecting the arm to a plastic block that slides along two parallel rods. A section of hollow aluminum tubing mounted to the slider block allows insertion of replaceable Teflon inlet tubing, with a small threaded hole and set screw in the slider block securely holding the tubing in place. The inlet tubing, cut at a steep angle (~45°) at the tip, can reliably puncture a thin plastic film sealing each well on the multiwell plate (Glad Press’n Seal). A complete online computer aided design (CAD, SolidWorks 2016) of the robot is available on GitHub^23^.

### *C. elegans* maintenance

The integrated *C*. *elegans* strain NZ1091 was developed by irradiation of CX16573^24^ and backcrossed at least 10X. Animals were grown and maintained at 20 °C on Nematode Growth Medium (NGM) plates with OP50 *E*. *coli* bacteria^25^. For the dose response experiment, *C*. *elegans* were selected as L4s the day before and tested as young adults the next day. For the chemical screen experiment, L4-stage *C*. *elegans* were picked and transferred to 50 µM All Trans Retinal (ATR) in OP50 *E*. *coli* spotted (150 µL) onto an unseeded 60 mm NGM plate 12 hrs prior to experimentation. The following strains were used:

NZ1091, kyIs587 [gpa-6p::GCaMP2.2b; unc-122p::dsRed]; kyIs5662 [odr-7p::Chrimson:SL2:mCherry; elt-2p::mCherry], expressing GCaMP and Chrimson in AWA neurons,

CX14887, *kyIs587* [*gpa-6*p::*GCaMP2*.*2b*; *unc-122*p::*dsRed*], expressing GCaMP in AWA neurons,

CX10979, *kyEx2865* [*sra-6*p::*GCaMP3*; *unc-122*p::*GFP*], expressing GCaMP in ASH neurons.

### Experimental control and automation

A suite of Arduino and MicroManager scripts automate experimental timing, serial liquid delivery patterns, stimulation controls, and image acquisition. A MicroManager graphical user interface (GUI) script enables user configuration of camera and illumination settings (exposure time, illumination pulse duration and delay, and image binning), multiwell plate positions, and timing of stimuli (Supplementary Fig. S2). These settings are transmitted *via* serial commands to two Arduino Uno microcontroller boards (Fig. 1b, Supplementary Fig. S2), one controlling the multiwell plate position and a second (Nobska Imaging) that sends digital pulses to control illumination and stimulation (e.g. microfluidic valves, optogenetic LED pulses, or others). With this setup, the same experiment settings (recording and stimulation) can be executed repeatedly for each well position. The robotic system can also function independently through customizable Arduino, MATLAB, or MicroManager code suites.

An Arduino program loaded on the robotic controller defines the well plate positions, motor steps per position, servo arm range, and timing of movement. Different settings allow adjustment to other well configurations (6- to 384-well plates) and plate depths (5 to 30 mm, or 300 µL to 2 mL for a 96-well plate). In its typical configuration, the Arduino code reads in serial command strings to control the plate position and tubing position (servo arm). For example, the ‘homed’ state (command: ‘0’) raises the servo arm and sets the plate to well A1. The command syntax is [*wellRow*][*wellColumn*][+ *or* −], which raises the servo arm, sends the plate to the specified well, and either keeps the servo up (+) or lowers it down (−) at the final position. Commands can be strung together with a semicolon delimiter for immediate sequential execution. For example, the string ‘A1-;A2-’ moves the plate to well A1, lowers the servo arm and tubing (‘−’), then immediately raises it again, moves to well A2, and lowers the tubing once again. The system remains in this configuration until another serial command is received (Supplementary Video S1).

For repetitive multiwell microscopy applications, two Micromanager scripts *GUI* and *RUN* can be used to coordinate robotic plate and tubing positions with fluidic valves, stimulation, and microscope image capture. *GUI* sets up the experiment parameters, and *RUN* executes them (Supplementary Fig. S2). A third script, *ROBOT*, offers manual control of multiwell plate position and valve states and can also initiate a sequence of well positions with independent durations per well (Supplementary Fig. S2). The *GUI* and *RUN* scripts were used for neural imaging (Figs 1–3) and *ROBOT* for the cell staining procedure (Fig. 4). These scripts can be configured to save time-stamped images and all settings metadata in a text file for automation of data analysis.

Alternatively, any program that sends serial commands can control the robotic system. For example, a MATLAB script^23^ automatically generates and transmits commands to move the plate from one well to another in a zigzag “snake” or “typewriter” pattern. Together, the Arduino code allows for fine adjustments to the robotic system, such as to configure different plate sizes and volumes, whereas MicroManager or MATLAB scripts define the sequences of well positions, timing for each experimental protocol, and data acquisition parameters. All software is available online at GitHub^23^.

### Bubble-free well-to-well transfer of inlet tubing

A backpressure reservoir connected to the microfluidic device applies a small positive hydrostatic pressure relative to the multiwell plate by elevating its fluid surface a few centimeters above the fluid surfaces in the plate. During well position switching, the outflow valve is closed and the backpressure valve is opened (Fig. 1c). The backpressure is adjusted by raising or lowering the bp reservoir such that no bubble enters the inlet tubing (bp too low) and no droplet forms at the inlet tubing (bp too high), which could transfer a small amount fluid to the next well (Fig. 1d). Once the robotic system positions the inlet tube into the next well, the backpressure valve is quickly closed, the outflow valve is opened, and the next fluid begins to fill the robotic inlet tubing and flow through the microfluidic device (Fig. 1c,e, Supplementary Fig. S3).

### Microfluidic device preparation

Single layer silicon master molds were designed using DraftSight and fabricated in the WPI MicroFabrication Laboratory cleanroom. Microfluidic devices were prepared from PDMS (Sylgard 184) and sealed reversibly to hydrophobic glass or permanently bonded, as previously described^26^. Devices were permanently-bonded to standard 1 mm thick glass slides for carryover and *in vitro* experiments, and reversibly-sealed to fluorinated glass slides for *C*. *elegans* neural response experiments. Device designs are available by request.

### Assessment of well-to-well carryover using fluorescent dye

A medium-depth 96-multiwell plate was prepared with 600 µL of water or 5 µg/mL fluorescein in alternating wells following a zigzag “snake” pattern and positioned on the robotic plate holder (Fig. 1f). A bonded microfluidic device with three ports was connected to inlet (in), backpressure (bp), and outlet (out) tubes, and flow was balanced by adjusting the height of the backpressure reservoir as described above. The inlet tubing was filled backwards by raising the buffer reservoir above the multiwell plate before rebalancing backpressure and inserting it into the first well. TIFF stack movies were acquired with a Hammamatsu Orca Flash 4 sCMOS camera and a 4×/0.28 NA Olympus objective mounted on an ASI RAMM microscope frame. Videos were recorded for 15 seconds per well at 10 fps with 2 × 2 binning and a 10 ms pulse of blue light (EGFP filter set, 470 nm excitation) from a Lumencor SOLA source that illuminated the fluorescein solution for each captured image frame. A 30 s fill delay was used between well switching. Mean integrated intensity was determined using ImageJ for each well in a 25 × 25 pixel region of interest at the convergence of microfluidic inlet channels, averaging the first, middle, and last frames per well to reduce noise. MATLAB was used to calculate carryover percent using these values by subtracting camera baseline (intensity of A1) from all values, then dividing the average baseline-corrected water well intensity by the immediately previous fluorescein well intensity (Fig. 1g,h).

To assess carryover with washing, a deep 96 well plate was prepared with 2 mL of water in each well and 2 mL of 5 µg/mL fluorescein in well A2. Data acquisition and analysis were performed as above.

### Calcium imaging, optogenetic stimulation, and data analysis

Calcium imaging was performed as previously described^17^. Microfluidic neural imaging devices were modified for 4X magnification (~2.5 mm arena size) and contained one or two physically-separated arenas (Figs 2 and 3, respectively). Devices quickly switch the chemical environment between a stimulus inlet stream and a buffer inlet stream by opening one of two control inlets (Supplementary Video S2, Fig. 2b). Fluorescence microscopy was performed using an ASI RAMM microscope as described above and configured for low-magnification wide-field imaging of GCaMP fluorescence. For optogenetic activation of the Chrimson cation channel, a 617 nm 3 W LED (Mightex) was mounted beneath the RAMM stage, filtered with a 645/75 nm bandpass excitation filter, and reflected to the objective with a 590 SP dichroic which also passed green GCaMP emission light to the camera (Fig. 3a). For both calcium imaging experiments, neuron fluorescence was quantified using the NeuroTracker ImageJ macro^17^ with a 6 × 6-pixel square for calculating integrated intensity, and custom MATLAB scripts for data aggregation, analysis and visualization. Contrast of wide-field images containing animals in the device was auto-adjusted for image print clarity using ImageJ in Figs 2d and 3b.

### Statistical comparisons

IBM SPSS was used for statistical tests, and significant suppressors of neural activity (Fig. 3d,e) were determined using a general linear model repeated measures analysis of variance (ANOVA). Significant p-values were selected based on solvent concentrations compared to immediately prior buffer control and noted in the figure after Bonferroni’s correction for multiple comparisons (*p < 0.001, Fig. 3e). Treatment effects were also assessed by one sample paired t-tests comparing the solvent response peaks minus prior buffer control peaks to a fixed zero value (no effect), yielding similar results after Bonferroni’s correction (*p < 0.001, Fig. 3f).

### Odor step-response in multiple *C. elegans* sensory neurons

Diacetyl (2,3-butanedione, Sigma) odor dilutions were prepared on the day of the experiment beginning with a 30 mL 1:1000 (10^−3^, 11.5 mM) dilution vortexed for 1 min in paralysis buffer (S. Basal buffer without cholesterol containing 1 mM acetylcholine agonist (–)-tetramisole hydrochloride). Serial dilutions were prepared in 10-fold steps until 11.5 nM (10^−9^) in a deep 2 mL glass-coated 96-well plate (WebSeal Plate+) and sealed with adhesive film (Glad Press’n Seal) (Fig. 2a). About twenty animals of each genotype were loaded into separate arenas and exposed for 1 hr to paralysis buffer from the buffer reservoir (Fig. 2d). During exposure, both buffer and outlet valves were open, and the odor stimulus inlet tubing was blocked. After animals were immobilized, tubing was inserted into the inlet port and allowed to fill backwards by raising the buffer reservoirs above the multiwell plate to elevate backpressure (>bp, Fig. 1d), then returned to the balanced position (=bp). The multiwell plate was moved to position A1, and flow patterns were checked for a proper step-change stimulus by control fluorescein dye buffer pulses. Calcium imaging was performed as described above, and automated acquisition began after negative control conditions (paralysis buffer containing 100 ng/mL fluorescein) were verified to show no neural response. At each well position, animals were exposed to buffer during the programmed 45 s fill delay, then presented with four 10 s odor pulses, once per minute. Image stacks were acquired for 30 s for each odor pulse at 10 fps with 10 ms blue light excitation pulse and 2 × 2 binning. After the experiment, the glass-coated multiwell plates were washed for reuse by completely filling and rinsing with water 3X, soaking in ethanol overnight, then rinsing with water again 3X, and drying in a 65 °C oven. Inlet and outlet tubing were also reusable following a water rinse step immediately after experimentation.

### Screen for solvent effects on *in vivo C. elegans* neural responses

Fourteen solvents were prepared in Eppendorf tubes at 1% and 5% v/v (or w/v for solids) concentrations in 1 mL paralysis buffer, vortexed for 1 min, and transferred to a medium-depth 96-multiwell plate (700 µL/well). Alternating control wells were filled with paralysis buffer (with 100 ng/mL fluorescein) to confirm baseline responses and visualize solution delivery (Fig. 3a). The 96-well plate was prepared 1 hr before recording and sealed with adhesive film (Glad Press’n Seal). During this time, animals were exposed to paralysis buffer *via* the backpressure reservoir to keep them stationary during neural recording as described above. Animals were exposed to each solvent for <1 min total, including a 30 s fill delay that was programmed to sufficiently fill the tubing and microfluidic arena after each well transfer. After this delay, an immediate 15 s acquisition began at 10 fps, with 5 ms blue light (470 nm) excitation and 2 × 2 binning. A single 5 s red light activation stimulus (617 nm, 38 mW/cm^2^) was presented from 2.5 to 7.5 s.

### Microfluidic cell culture

Human mesenchymal stem cells (hMSCs, P7-P8) were grown to confluency at 37 °C with 5% CO_2_ in standard growth medium (hMSCgm bullet kit, Ionza). The cells were washed, trypsinized, centrifuged, and re-suspended with fresh growth media to approximately 5 × 10^6^ cells/mL. A cleaned and bonded PDMS device, containing a long serpentine channel, was prepared by baking at 65 °C for ~2 hrs and degassing in a vacuum desiccator for 15 min. Next, a drop of growth medium was placed over the inlet and drawn into the microfluidic channel by a hand-held pipette inserted into the outlet (Fig. 4a). The cell suspension was then drawn through the device in the same manner, and the device was placed in an incubator to settle and attach to the glass surface overnight. The next day, the cell-loaded device was placed on the microscope stage (at room temperature) and connected to all tubing inlets and outlets as previously described (Fig. 4b). The backpressure phosphate-buffered saline (PBS) solution was used to fill the robotic inlet tubing by gravity driven flow as described above before lowering it into the first multiwell plate position.

### Automated cell fixation and staining

Staining solutions were prepared in PBS at 2 mL final volumes and loaded into the first 10 wells (A1-A10) of a deep (2 mL) 96-well plate (WebSeal Plate+). The solutions were 4% w/v paraformaldehyde (PFA), 0.25% v/v Triton X-100, 1% w/v bovine serum albumin (BSA), 2.5% v/v Alexa Fluor 488 (AF488) phalloidin (ThermoFisher A12379), and 0.0167% v/v Hoechst 33342 (Fig. 4c). At each well position, a 30 s delay was programmed to sufficiently switch from one inlet solution to the next. The staining procedure was performed in the following sequence: 30 s PBS wash, 10 min fixation in 4% PFA, 30 s PBS wash, 10 min permeabilization in 0.25% Triton X-100, 30 s PBS wash, 30 min blocking in 1% BSA, 30 min f-actin staining with 2.5% AF488 phalloidin, 30 s PBS wash, 3 min nuclear counterstain with 0.0167% Hoechst, and a final 30 s wash in PBS. A TIFF image stack of the device channels with cells was recorded at 1 frame per minute for 90 min throughout the automated staining protocol using a pulse of blue light during each acquisition (Supplementary Video S3). ImageJ was used to quantify change in green fluorescence across all 90 frames of one representative microfluidic channel (Fig. 4d). After staining, cells were sealed to prevent drying by applying Cytoseal to all inlets and outlets and stored at 4 °C. After staining, higher magnification images of the stained cells were obtained on an inverted Leica fluorescent microscope with FITC and DAPI filtered images overlaid using ImageJ (Fig. 4e-g). Contrast was auto-adjusted for image print clarity using ImageJ (Fig. 4f-g). The remaining staining solutions in the multiwell plate could be reused multiple times when stored at 4 °C and sealed.

### Data availability

All software, scripts, design files, and an interactive visualization of chemical screen data is made available on GitHub^23^, and all raw data and CAD design files are available upon request.

## Electronic supplementary material

Supplementary Information

Supplementary Video 1

Supplementary Video 2

Supplementary Video 3
