# Supplementary material for: Automated fluid delivery from multiwell plates to microfluidic devices for high-throughput experiments and microscopy
Source: Sci Rep. 2018 Apr 18;8:6217. doi: 10.1038/s41598-018-24504-x (PMC5906459; doi:10.1038/s41598-018-24504-x)
Supplement: Supplementary file 1 — Supplementary Information [file 41598_2018_24504_MOESM1_ESM.pdf]

Automated fluid delivery from multiwell plates to microfluidic devices for high-throughput experiments and microscopy

Ross C. Lagoy, Dirk R. Albrecht

Supplementary Information

Supplementary Video S1. A multi-angled spliced video of the robotic platform and complete system functioning autonomously after initial microfluidics setup, positioning of the multiwell plate, and configuration of experimental settings. Video is edited from multiple camera views in real-time or accelerated as noted on-frame.

Supplementary Video S2. A wide-field reversibly-sealed microfluidic device demonstrating the pulsed flow switching dynamics. The device contains four inlets: the stimulus inlet ('in' containing fluorescein), a buffer inlet, and two valve-actuated control channels (c_1 and c_2 containing lower-concentration fluorescein for visualization). Only one control channel flows at a time, directing either the stimulus or buffer into the monitored worm arena chamber(s). Full video is ~11 mm x 3 mm in real-time (10 fps) as noted on-frame in seconds.

Supplementary Video S3. A wide-field bonded microfluidic device with hMSCs seeded into a serpentine channel, imaged every 1 minute for 90 minutes to capture changes in fluorescence (470 nm excitation) throughout the entire staining protocol. One channel is 200 µm in width, and video is 60X real-time (original 1 fpm) as noted on-frame in minutes.

Supplementary Table S1.

	**Our Robotic System**	**Rotary Valve**[1] (Hamilton MVP)	**HPLC Autosampler**[2] (UltiMate WPS-3000)	**Autosampler**[3] (Aurora AS100)
**Liquid delivery technique**	Inlet tubing transfer	Rotary valve	Needle/syringe injection	Needle/syringe injection
**Number of reservoirs**	Up to 384	Up to 8 or 12	> 384	Up to 1536
**Reservoir volume**	225 µL (384 well)<br>2.2 mL (96 well)<br>16.8 mL (6 well)	~1 mL – >1 L	10 nL – 125 µL	100 nL – 1 mL
**Mechanical switch time**	~2 s	~0.4 s	n.r.	n.r.
**Fluid switch time** (in device, at 2 µL/s)	~30 s	~30 s	not possible (max 0.03 µL/s)	n.d.
**Carryover***	<0.02%	n.d.	<0.02%	n.r.
**Approximate cost**	<$500	$1,300	>$8,500**	$12,000
**Format and size** (h x w x d)	Fits on microscope stage, 19x45x31 cm^3	Small unit, 10x6x10 cm^3	Large frame, 36x42x51 cm^3	Large frame, 16x52x58 cm^3
**References (use with microfluidic devices)**	this work	17, 25		10

Supplemental Table S1. Comparison of liquid delivery systems from multiple wells or reservoirs to microfluidic devices. Some values are not reported (n.r.) or not determined (n.d.). Mechanical switch time refers to the transfer of tubing, needle, syringe, or rotation of a valve. Fluid switch time refers to the exchange of liquid within the microfluidic device. References are from the main article.

Notes:

* Carryover estimate by measurement of caffeine (UltiMate 3000) or fluorescein dye (our robotic system).

** Cost reported for a used unit.

Instrument references:

[1]Rotary Valve, Hamilton Company Serial Modular Valve Positioner (MVP). *OEM Liquid Handling Components*. Valve Positioner. Retrieved from: https://www.hamiltoncompany.com/products/liquid-handling-components/valve-positioner/serial-mvp on March 16, 2018.

[2]HPLC Autosampler, Thermo Scientific Dionex UltiMate 3000 RSLCnano System. *Product Specifications*. Key Autosampler Specifications. pg. 6. Retrieved from: https://assets.thermofisher.com/TFS-Assets/CMD/Specification-Sheets/PS-70195-LC-Dionex-UltiMate-3000-RSLCnano-PS70195-EN.pdf on March 16, 2018.

[3]Autosampler, Aurora Universal XYZ Autosampler. OEM Components. Retrieved from: http://www.aurorabiomed.com/wp-content/uploads/2014/02/OEM-autosampler.pdf on March 16, 2018.

Supplementary Figure S1.

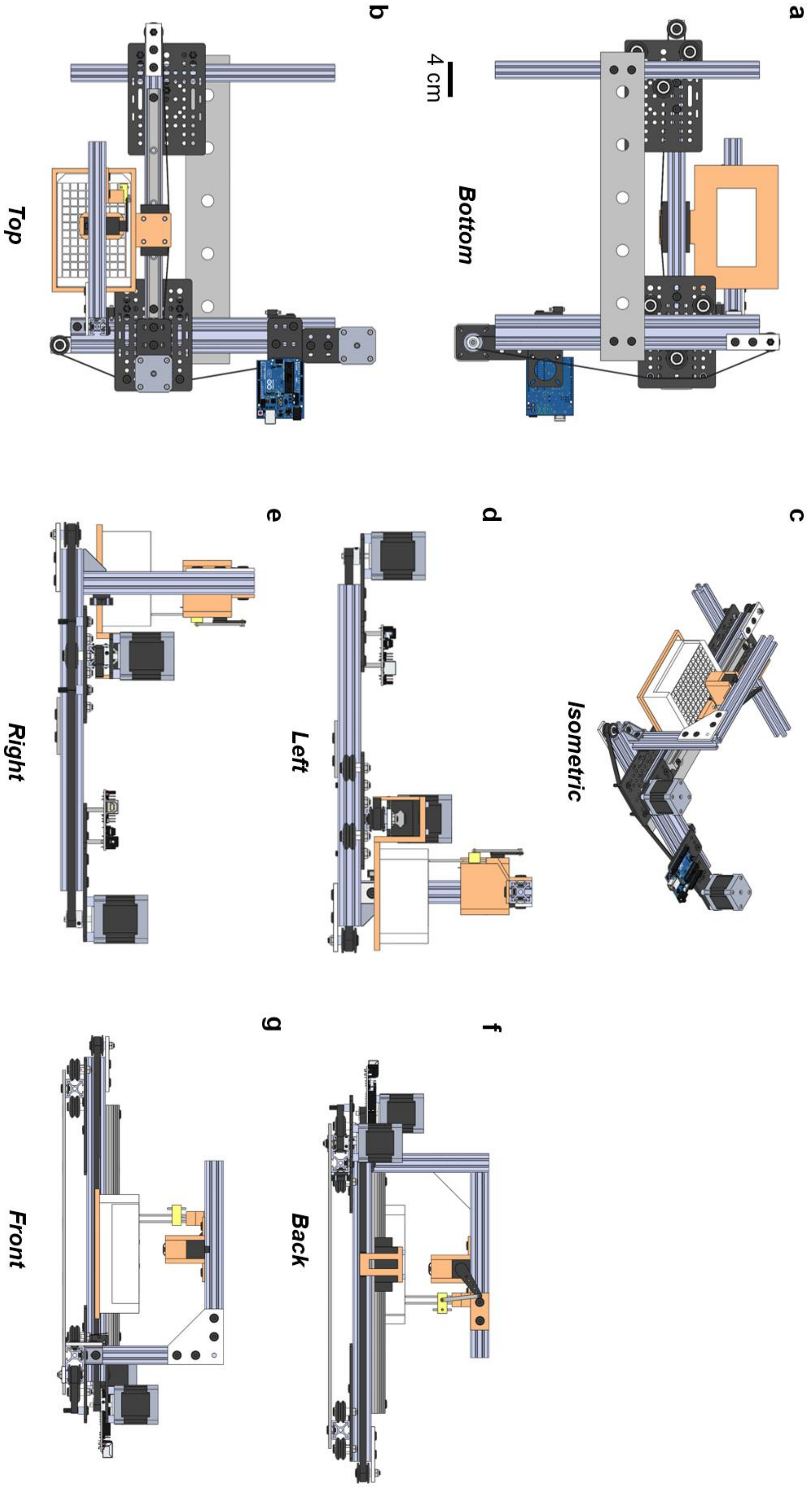

Supplementary Figure S1. Multiple angle views of the modular robotic system design.

a. A bottom view of the robotic system showing versatility of microscope stage mounting with an aluminum bar of equally spaced, large diameter mounting holes.

b. A top view of the robotic system showing the range of x-y motion on the linear slide rail and v-slot rails. Both motors are mounted vertically, with belts positioned through v-slot rails or fastened to the multiwell plate holder (orange). Two v-slot universal plates are used to mount the linear slide rail with standard hardware. The servo linkage assembly is aligned with the A1 'home' position in the top left (as shown in all panels). An Arduino R3 Uno is mounted towards the back-right, protected by a limit switch.

c. An isometric view of the robotic system showing both x-y motors in front, and the servo linkage assembly mounting arm using cut v-slot rails and mounting hardware.

d. A left-side view of the robotic system showing the alignment of the multiwell plate and servo linkage assembly in the raised 'up' position (as show in all panels), with motors to the far side. The multiwell plate holder (orange) is elevated above the left v-slot rail, allowing for full x-range and removal of the multiwell plate. The multiwell plate is also connected to the x-range drive belt.

e. A right-side view of the robotic system showing the alignment of the y-range belt and front of the servo linkage assembly, similar to d.

f. A back-side view of the robotic system showing the servo linkage assembly aligned. The multiwell plate is elevated above the bottom aluminum bar to ensure sufficient spacing from all moving parts with various microscope stages and mounting platforms.

g. A front-side view of the robotic system showing the alignment and height of the servo linkage with the top of the multiwell plate. The height of the servo mount can be adjusted by a T-nut for use of various depth multiwell plates (2 mL depth shown here).

Supplementary Figure S2.

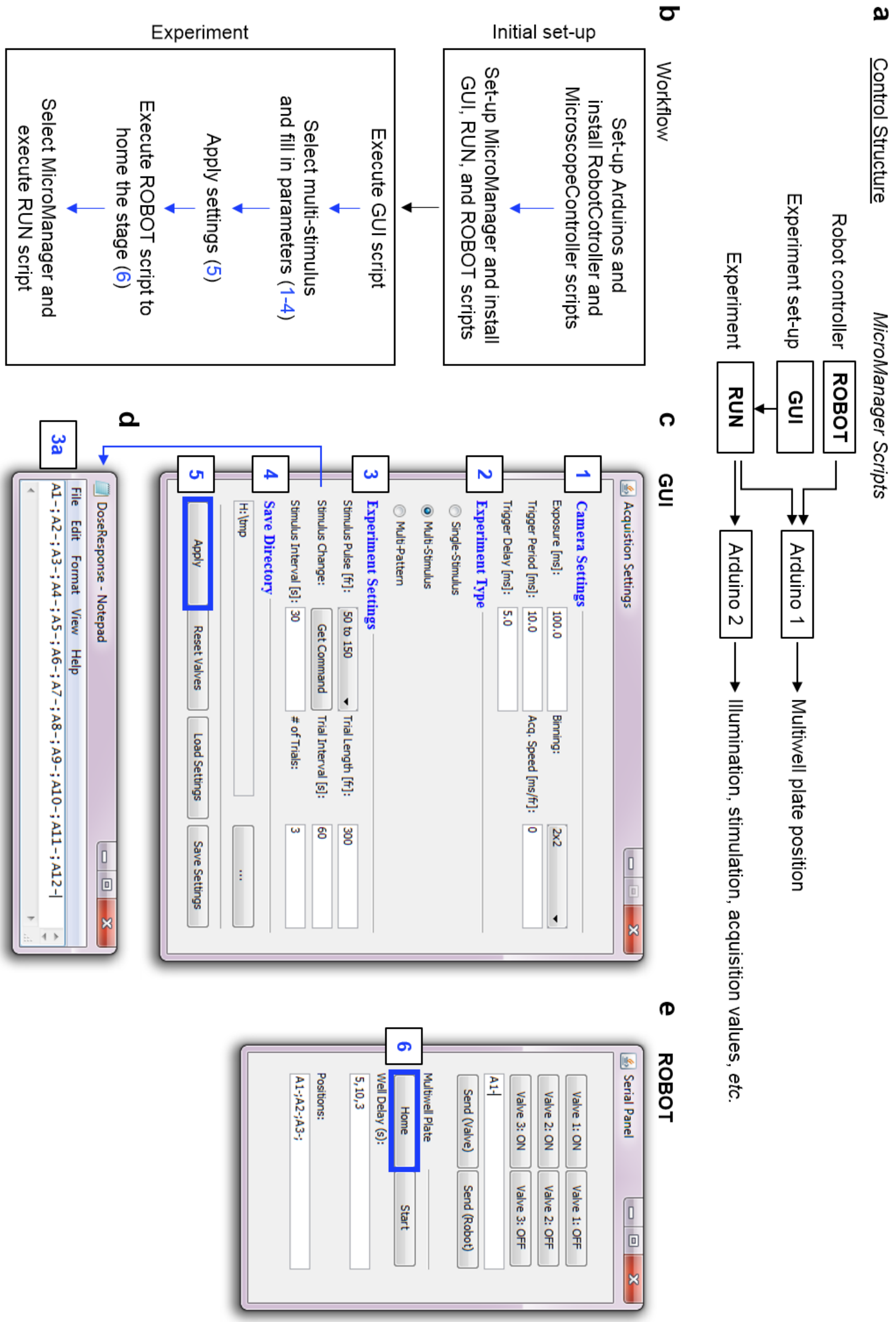

Supplementary Figure S2. Software control structure and custom graphical user interfaces (GUIs) developed in MicroManager enabling input of experimental settings and automation of the robotic system.

a. Simplified flow diagram of *GUI*, *RUN*, and *ROBOT* control scripts developed in MicroManager for independent robot automation *via* Arduino 1 and for control of stimulation, valves and image capture *via* Arduino 2.

b. Flow diagram of initial set-up (upper box), and experiment execution steps (lower box). Numbers correspond to selections in the user interface shown in panels c-e.

c. A screen shot of the Acquisition Settings GUI that allows user input for Camera Settings: (1) *Exposure*, (2) *Trigger Period*, and (3) *Trigger Delay*. The Experiment Type *Multi-Stimulus* corresponds to robotic experiments with the same timing and pulse pattern per well, as defined in Experiment Settings. *Stimulus Pulse* is used to set the state [on/off] of one stimulation valve used for microfluidic pulse experiments. *Stimulus Interval* sets the fill delay (t_{fill}), or time to wait until the next stimulus has completely filled the inlet tubing. *Trial Length* allows the user to specify how long to record the video in frame count. *Trial Interval* corresponds to the time between repeated trials. The *# of Trials* defines the number of repeated trials per well. (4) *Save Directory* indicates where imaging data is stored. (5) Settings are saved and finalized by clicking *Apply*.

d. A sample text file with well plate and servo positions for the *Stimulus Change* option for all wells in the first row, from c.

e. A second GUI called *ROBOT* is used for independent control of position, timing, and valve state [on/off]. The first box allows the user to manually enter the single well position for the robot to move to, as well as the servo arm position once it reaches that well. The robot can be 'homed' to A1 (below). A comma separated list of well duration times (in seconds) can be entered to the *Well Delay* box, each corresponding to a semicolon separated string of multiwell plate positions and servo states in the *Positions* text box, with a syntax described in Methods. TIFF stacks are acquired by applied camera settings using *GUI* and saved per set well position and timing after clicking *Start*.

Supplementary Figure S3.

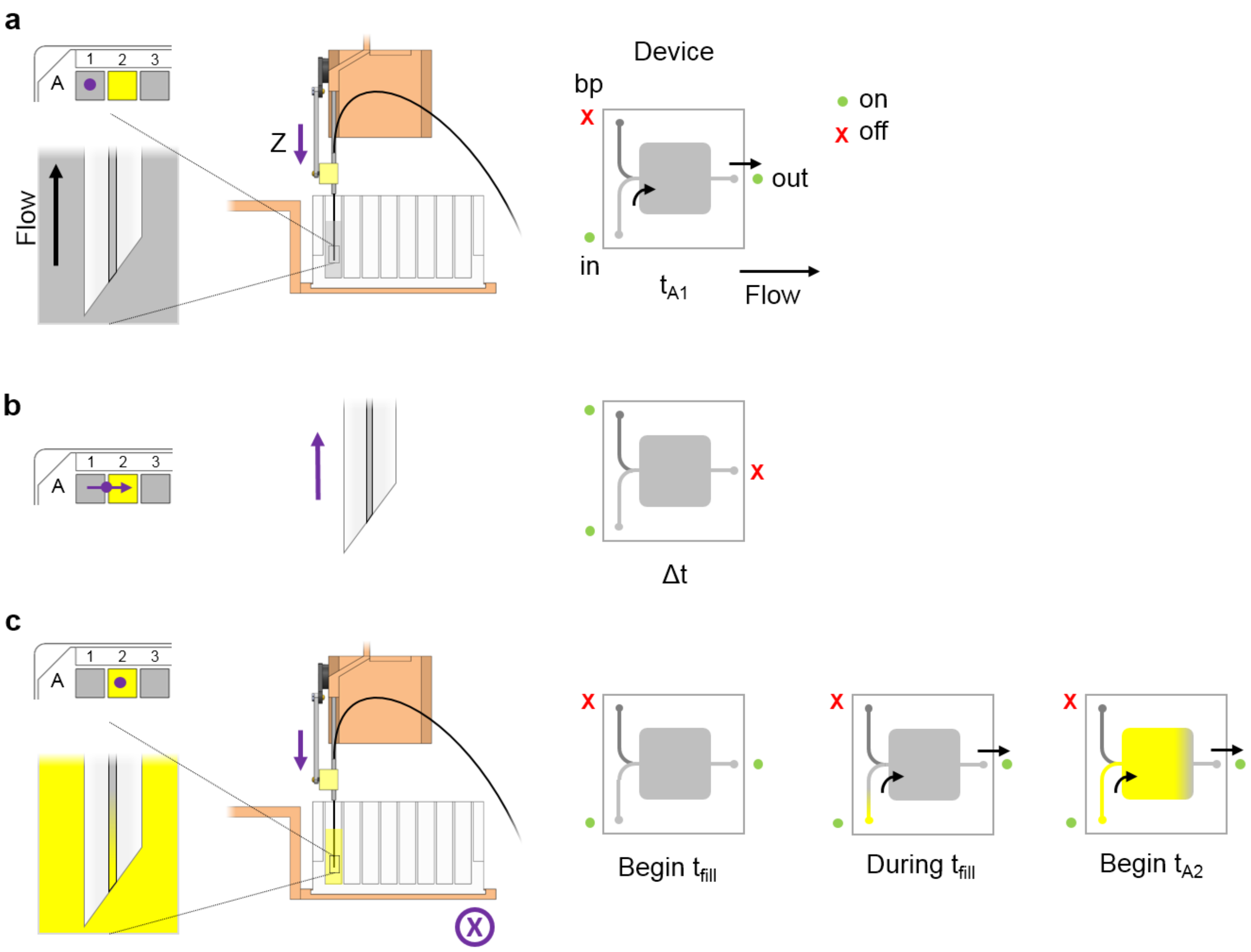

Supplementary Figure S3. Schematic of tube positions, valve states, and microfluidic flow before, during, and after a stimulus transition.

a. Before the stimulus transition, the servo arm lowered the tubing tip into liquid (gray) of well A1, shown as a purple dot in the top-view of the multiwell plate. A magnified view of the tubing tip shows a full tubing channel containing A1 well fluid (gray). The outlet (out) valve state is 'on' and backpressure (bp) is 'off', allowing fluid to flow through the microfluidic device from well A1.

b. During the transition of well plate position from A1 to A2, the tubing tip raises up by the servo arm. No air enters the tubing because the outlet valve is 'off', and the backpressure valve is turned 'on'.

c. The multiwell plate is then positioned to well A2 (yellow), the tubing tip is lowered into the well, backpressure is turned 'off', and the outlet is rapidly turned 'on' to restore fluid flow. During the initial t_{fill} state, only the prior well fluid (A1, gray) flows through the microfluidic device, while the new fluid (A2, yellow) begins to fill at the inlet tip channel. A short time later, the current well fluid begins to fill the microfluidic inlet channel and device with the next solution (yellow). The required t_{fill} time is inversely proportional to flowrate, about 30 s at 2 µL/s.

Supplementary Figure S4.

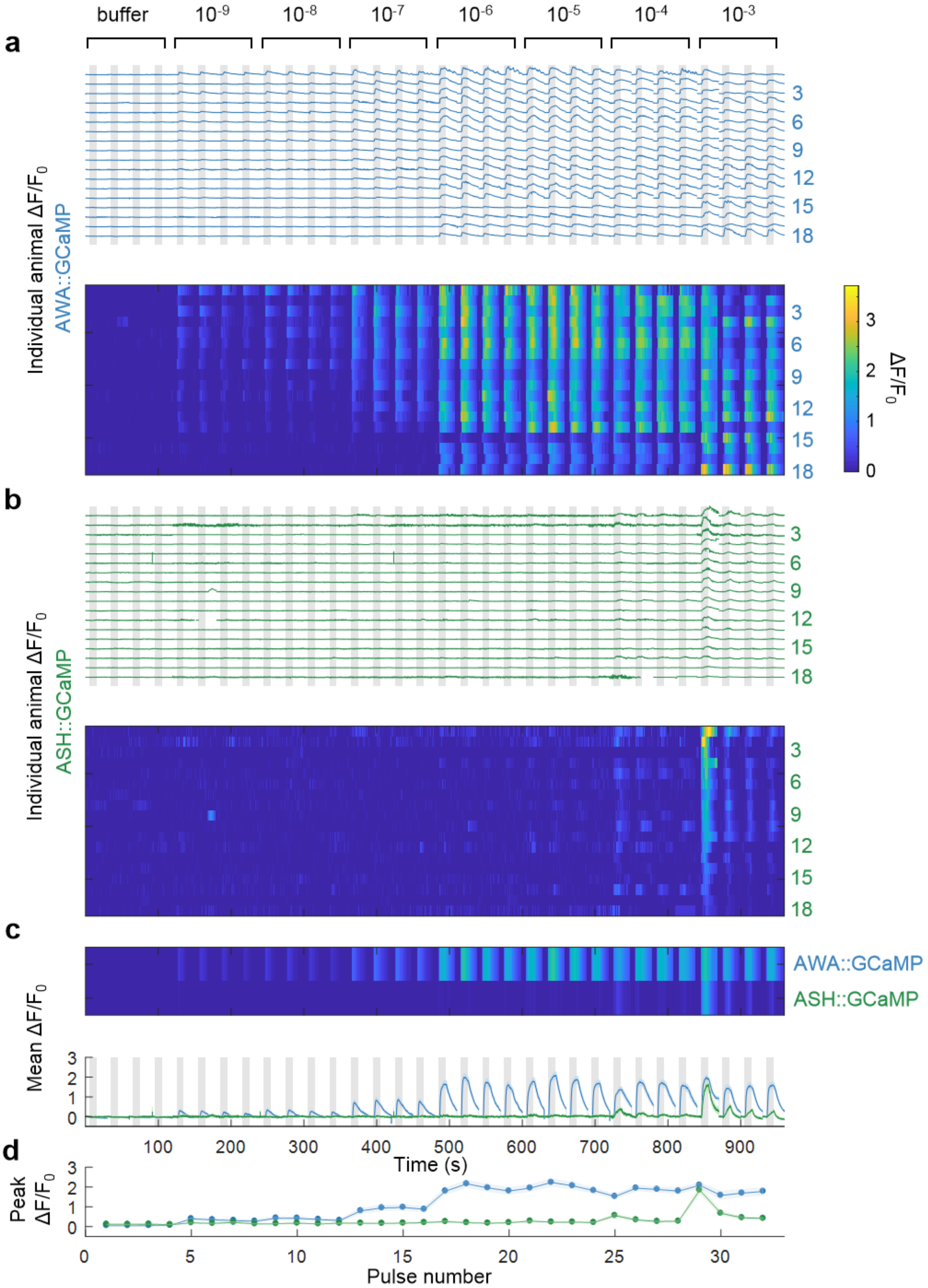

Supplementary Figure S4. Calcium dose-response summary across multiple pulses, concentrations, and neurons in an automated odor step response experiment.

a. Individual $\Delta F/F_0$ calcium response traces for 18 AWA::GCaMP animals across all pulses (gray shading) and concentrations (first four buffer, then 11.5 nM to 11.5 mM with 10 fold step increases). Traces are sorted in descending order by the first pulse max response to 10^{-7}, or 1.15 µM. A corresponding heat map of individual responses is shown below.

b. Individual $\Delta F/F_0$ calcium response traces for 18 ASH::GCaMP animals, and sorted in descending order by the first pulse max response to 10^{-3}, or 11.5 mM. A corresponding heat map of individual responses is shown below.

c. Population-average fluorescence $\Delta F/F_0$ calcium responses by neuron (AWA::GCaMP above, ASH::GCaMP below) across all pulses and concentrations corresponding to timing above, shown as a heat map (above) and mean traces (below). Shading represents SEM.

d. Mean peak $\Delta F/F_0$ calcium responses by genotype across all pulses and concentrations corresponding to c. Shading represents SEM.

Supplementary Figure S5.

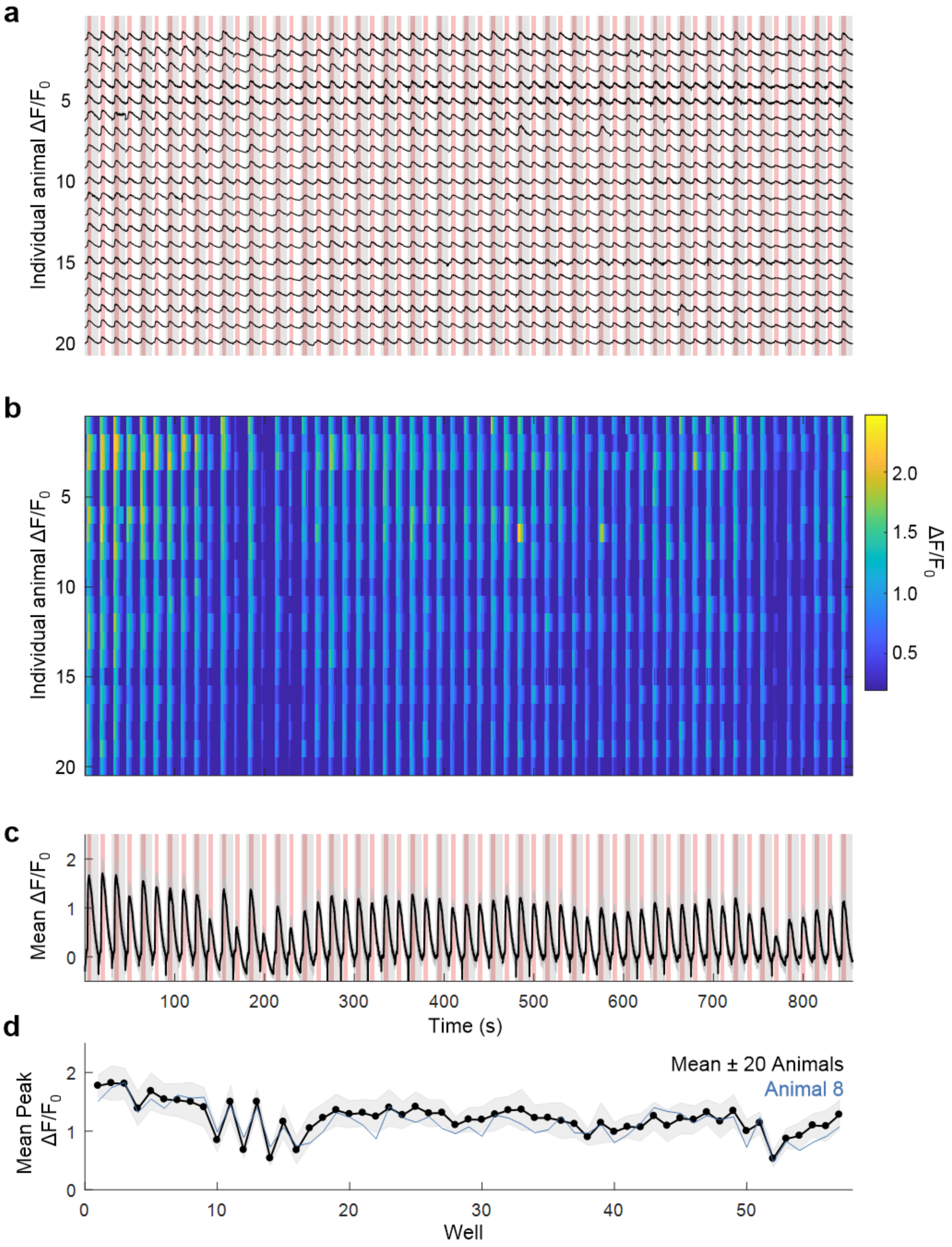

Supplementary Figure S5. Calcium responses to optogenetic activation pulses in different buffers, solvents, and carriers.

a. Individual $\Delta F/F_0$ calcium response traces for animals co-expressing GCaMP and Chrimson in AWA sensory neurons (n = 20) across all 57 red-light pulses (red shading, 38 mW/cm^2) and 57 liquid delivery wells (alternating buffer-solvent concentrations, gray shading represents buffer wells). Traces are sorted in descending order by well 11 (buffer).

b. Corresponding heat map to a.

c. Averaged $\Delta F/F_0$ calcium responses (black line) from 20 animals across all 57 pulses and liquids delivered, corresponding to individuals in a and b. Shading represents SD.

d. Mean peak $\Delta F/F_0$ calcium responses (black line) corresponding to c. One individual animal (#8) is shown by the blue dots and line. Shading represents SD.
